# The role of feedback and differences between good and poor decoders in a repeated word reading paradigm in first grade

**DOI:** 10.1007/s11881-016-0129-z

**Published:** 2016-04-11

**Authors:** Karly van Gorp, Eliane Segers, Ludo Verhoeven

**Affiliations:** Behavioural Science Institute, Radboud University Nijmegen, P.O. Box 9104, 6500 HE Nijmegen, The Netherlands

**Keywords:** Direct, retention, and transfer effects, Feedback effects, Good and poor beginning readers, Repeated word reading

## Abstract

The direct, retention, and transfer effects of repeated word and pseudoword reading were studied in a pretest, training, posttest, retention design. First graders (48 good readers, 47 poor readers) read 25 CVC words and 25 CVC pseudowords in ten repeated word reading sessions, preceded and followed by a transfer task with a different set of items. Two weeks after training, trained items were assessed again in a retention test. Participants either received phonics feedback, in which each word was spelled out and repeated; word feedback, in which each word was repeated; or no feedback. During the training, both good and poor readers improved in accuracy and speed. The increase in speed was stronger for poor readers than for good readers. The good readers demonstrated a stronger increase for pseudowords than for words. This increase in speed was most prominent in the first four sessions. Two weeks after training, the levels of accuracy and speed were retained. Furthermore, transfer effects on speed were found for pseudowords in both groups of readers. Good readers performed most accurately during the training when they received no feedback while poor readers performed most accurately during the training with the help of phonics feedback. However, feedback did not differentiate for reading speed or for effects after the training. The effects of repeated word reading were found to be stronger for poor readers than for good readers. Moreover, these effects were found to be stronger for pseudowords than for words. This indicates that repeated word reading can be seen as an important trigger for the improvement of decoding skills.

It is crucial that orthographic representations are being stored and that word reading becomes automatized, since fluent word reading plays a very important role in text reading fluency (Torgesen, Rashotte, & Alexander, 2001) and comprehension (Perfetti & Stafura, 2014). When beginning readers encounter words, they use phonological recoding, i.e., graphemes are recoded into phonemes. When children perform this phonological recoding process, they provide themselves with feedback on each successful encounter (Perfetti, 1992). By means of this feedback, the orthographic representation of a word becomes incrementally stronger. This process of phonological recoding can be seen as a self-teaching mechanism (cf., Share, 1995, 2004). Poor readers of orthographically transparent languages do not so much struggle with the phonological recoding process itself, but rather with automatization of this process (Wimmer, Mayringer, & Landerl, 1998). One way to overcome this automatization problem is to train these children using repeated word reading (Berends & Reitsma, 2006). However, even though repeated reading of words was found to be successful in improving both accuracy and speed of trained items, most studies either did not assess (e.g., Martens & De Jong, 2008), or failed to find transfer effects to untrained items (e.g., Berends & Reitsma, 2006). Moreover, although immediate corrective feedback is claimed to be crucial to obtain transfer effects in repeated reading of texts (Therrien, 2004), the role of feedback on transfer effects of repeated word reading has not yet been investigated. In the present study, we examined the effect of different types of feedback on training, retention, and transfer in a repeated word and pseudoword reading paradigm comparing good and poor readers in first grade.

Repeated word reading can be seen as a paradigm in which orthographic learning as a consequence of word repetition occurs in children as young as 7 years old (Reitsma, 1983). The word repetition effect has not only been demonstrated with isolated words but also in reading in context. In a study with young children reading novel words embedded in text, Share (2004) showed that for most children, a few exposures to an unknown letter string already is sufficient to store orthographic information about this string in their mental lexicon. In third graders, Share (2004) found the largest progress in repeated novel word reading between the first and second encounter of the word. This result is in line with the instance theory of automatization of Logan (1988). According to the instance theory, orthographic learning for normal readers starts directly with the first encounter of a word. Once stored, the orthographic information has shown to be retained 30 days (Share, 2004) or even 10 weeks later (Hogaboam & Perfetti, 1978). An alternative view on orthographic learning is the threshold model (e.g., Reitsma, 1983) according to which a certain number of encounters (typically three or four) is needed for orthographic learning to occur. However, for poor reading children it remains unclear how many exposures to an unknown letter string are needed to come to a stable orthographic representation. Lemoine, Levy, and Hutchinson (1993) presented poor reading children with the same set of English words five to 25 times. They found no continuing increase in reading speed after only six repetitions, which might reflect that orthographic learning has occurred within the first six repetitions. They did, however, find better retention effects if the words were repeated more often. In another study by Martens and De Jong (2008) in Dutch, it was found that after 20 repetitions of the same word, the word length effect did not disappear in poor readers. This showed that poor readers read longer words at a slower pace than shorter words, even after 20 repetitions.

The ultimate goal of repeated word reading is reaching transfer effects to reading untrained words. To date, only a limited number of studies focused on repeated reading of single words (see Wolf & Katzir-Cohen, 2001), and only few of these also assessed transfer to untrained items (e.g., Berends & Reitsma, 2006; Lemoine et al.,^1993^). Lemoine et al. (1993) conducted three experiments in which direct, retention, and transfer effects were examined in both good and poor readers in grade 3, and poor readers in grade 4. They found direct and retention effects. However, with regard to transfer, they did not succeed in inducing generalization effects, regardless of the amount of orthographic overlap between trained and untrained items and regardless of the amount of repetitions during training. Thaler, Ebner, Winmer, and Landerl (2004) studied the transfer effect of the inclusion of similar onsets in trained and untrained words in poor reading children in grades 2, 3, and 4. The children were presented with 32 training words, with eight different onsets. Each word was repeatedly read by the participants in an experimental setup with six presentations per session, for up to 25 days. There was only a small transfer to words containing the same onset and no transfer to orthographically unrelated words. Finally, Berends and Reitsma (2006) compared the effects of repeated word reading with those of single word reading. Half of the participants, who were poor readers, read the same set of 20 words 20 times, whereas the other half read 400 different words. Feedback regarding accuracy was given, but only by the presentation of a smiley. At posttest, trained items were read faster by the repeated word reading group. For both conditions, there were no transfer effects for untrained items. Even though the children in this latter repeated word reading study received feedback on the task, the learning gains were not at the expected level. A possible explanation is that this particular feedback only involves information about the correctness of the response and not about the correct pronunciation of the word. The accuracy of this group of readers was already close to 90 % at the start of the intervention. In order to improve reading fluency, they therefore might need feedback which helps them gain speed, rather than feedback on correctness alone. Even though accuracy in beginning readers of Dutch is rather high, the errors that are made are unstable (i.e., different rather than the same words are read incorrectly while read repeatedly) (Steenbeek-Planting, van Bon, & Schreuder, 2013). In all, in these repeated word reading studies, there was little or no transfer to untrained items.

Interestingly, transfer effects have been found for repeated text reading when including corrective feedback, i.e., feedback which indicates whether a response is correct or not (see Therrien, 2004). Feedback may thus also be important in obtaining transfer effects in repeated reading of words. Following the theory of Perfetti (1992) on phonological recoding, children provide themselves with feedback on each encounter of a word. For poor readers of transparent languages, however, this repetition alone is clearly not sufficient to reach the stage of automatic word reading (e.g., Martens & De Jong, 2008). In a meta-analysis, Hattie and Timperley (2007) evidenced that corrective feedback is particularly helpful while acquiring a new skill. Hattie and Timperley (2007) also found that the timing of feedback can be crucial. While acquiring a specific skill, immediate feedback can be helpful but while building fluency, immediate feedback can disturb the process of automatization. With regard to the agent providing the feedback, it turns out that computer-assisted feedback is among the most effective types, alongside video and audio feedback (Hattie & Timperley, 2007). Rasinski, Homan, and Biggs (2009) reported that feedback while acquiring reading fluency is important. When a word is read incorrectly, it is important that a correct representation is provided, because, otherwise, the reader might store the incorrect representation. Moreover, Rasinski et al. (2009) argued that, in addition to receiving feedback, students should listen to others reading fluently. Indeed, it has been found that learners are likely to follow the model in order to increase the chance of success and hence their self-efficacy (Schunk, 2003). In a similar vein, it has been shown that children’s self-efficacy can be improved, if feedback includes a suggestion for improvement rather than just an indication of whether the response was correct (Chan & Lam, 2010).

A few studies examined transfer effects of various types of corrective feedback in repeated reading of syllables or words. In two studies that incorporated immediate corrective feedback, including the correct representation, transfer effects were evidenced after repeated reading of syllables (Huemer, Aro, Landerl, & Lyytinen, 2010), or words and pseudowords (Van Gorp, Segers, & Verhoeven, 2014). The feedback that was used in both studies included information on correctness of the named items, after which the correct pronunciation was proved by either the experimenter or the computer. Huemer et al. (2010) focused on repeated reading of syllables in Finnish speaking children in grades 4 to 6. If an item was read incorrectly, the tutor asked the child to reread the word. When the word was read incorrectly twice, the tutor would provide the correct pronunciation. Huemer et al. found transfer effects from the syllables to multisyllabic pseudowords containing these trained syllables. In a recent study, we (Van Gorp et al., 2014) examined the effects of repeated word readings in emergent readers (i.e., kindergartners) comparing various types of feedback. Both word feedback (i.e., immediate corrective feedback on correctness followed by pronunciation of the word) and phonics feedback (i.e., immediate corrective feedback on correctness followed by pronunciation and spelling of the word) turned out to be effective for kindergartners. Both speed and accuracy increased and transfer effects for untrained items were evidenced. In this recent study, no differences were found between the two types of feedback, and it was also not examined what the effect of feedback was as compared to a condition without feedback. Transfer effects after repeated reading of words were thus obtained for emergent readers when they received feedback (Van Gorp et al., 2014). However, it was not investigated whether the same findings would be obtained without the inclusion of feedback. Moreover, the sample in the study of Van Gorp et al. (2014) consisted of good reading kindergartners who had not received any previous reading instruction.

An important remaining question is what the role is of feedback in repeated word reading among developing readers. The first step towards becoming an automatic reader is to be an accurate reader. Reaching high levels of accuracy takes more time in opaque languages than in transparent languages, in which readers make fewer errors (Aro & Wimmer, 2003; Seymour, Aro, & Erskine, 2003). It is assumed that beginning and more experienced readers make use of different strategies while reading words. Following the dual route cascaded (DRC) model of reading (Coltheart, Rastle, Perry, Langdon, & Ziegler, 2001), reading novel word strings and pseudowords occurs via the indirect or non-lexical route. In this route, words are decoded letter by letter. Familiar words are read via the direct or lexical route in which word recognition occurs automatically. As readers become more experienced at reading, the use of the direct route increases. Martens and De Jong (2008) investigated the influence of repeated word reading on direct or indirect reading of words as a function of word length. If orthographic learning would occur after repeated word readings, typical length effects which are thought to reflect letter-by-letter reading rather than direct access would disappear after a series of repetitions. This would also imply a shift from the indirect route to the direct route. However, Martens and De Jong (2008) found that after 16 repeated word readings without feedback, the length effect decreased in typically reading fourth and fifth graders but lasted in typically reading second graders and poor reading fourth and fifth graders. Even though reading speed increased over time, the authors concluded that poor and beginning readers still relied on the indirect route of reading. In a similar study, Suárez-Coalla, Ramos, Álvarez-Cañizo, and Cuetos (2014) showed that the length effect decreased in typically reading Spanish children (aged 7-12) but lasted in dyslexic children. The study of Martens and De Jong (2008) was on beginning readers of Dutch, an orthographically transparent language. They concluded that repeated reading of single words did lead to automatization in typically reading children, but not in poor readers. With regard to the DRC model, this implies that poor readers of Dutch (and other orthographically transparent languages (e.g., Spanish; Suárez-Coalla et al., 2014)) are thought to keep relying on the indirect route of reading (i.e., phonological recoding), whereas typically developing readers have automatized reading and move towards direct reading, at least after 16 repetitions.

In the present study, we focused on the role of feedback and differences between good and poor readers in a repeated word reading paradigm in Dutch first graders. The Dutch language can be seen as a relatively transparent language (Ziegler, Bertrand, Tóth, Csépe, Reis, Faísca, & Blomert, 2010). In previous research, it has been evidenced that at the end of first grade, mean accuracy scores for CVC words are just below 90% (Verhoeven & Van Leeuwe, 2009). In the research so far, it has often been evidenced that repeated reading of words is effective in increasing reading accuracy and speed of the trained words (for meta-analyses see: Kuhn & Stahl, 2003; Therrien, 2004). However, transfer to untrained items is often not occurring when no immediate corrective feedback is included. Two repeated reading studies that did include this type of corrective feedback did find transfer effects (i.e., Huemer et al., 2010; Van Gorp et al., 2014). Both studies did not include a condition without feedback, which is needed to ascribe the presence of transfer effects solely to the inclusion of immediate corrective feedback. In the present study a condition without feedback was included to examine whether this was indeed the crucial factor in the previous studies. In the present study, we further investigated the role of various forms of feedback (none, word, or phonics) on the growth of reading accuracy and speed in beginning readers. To our knowledge, feedback has been included in previous studies on repeated word reading, but it has never been established whether feedback indeed is effective. The present study will try to answer this question. Moreover, since it is found that beginning readers move from the indirect route of reading to the direct route of reading (e.g., Martens & De Jong, 2008) it could be that different types of feedback elicit different results. Hence, the phonics feedback corresponds more with the indirect route of reading, whereas the word feedback corresponds with the direct route of reading.

Furthermore, in previous research on repeated word reading with feedback (Van Gorp et al., 2014), good reading kindergartners were assessed before they had received formal reading education. The transfer effects that were found were likely caused by the inclusion of feedback. Another possibility might be that transfer effects were obtained because these children were good readers and were able to make use of analogies between the trained and untrained items (Savage & Stuart, 2001). In the study of Huemer et al. (2010), the mean age of the subjects was 11 years. As interventions should take place as soon as possible, we focused on 6-year-olds. In the present study, we also compared good readers to poor readers to examine the differences between these groups of readers. Good beginning readers are found to have good orthographic knowledge after a few encounters (e.g., Share, 2004). We wanted to examine how poor readers performed on the same task as compared to good readers. To our knowledge, this direct comparison has not been made. It might well be the case that good readers will reach the direct route of reading already within ten repetitions, whereas beginning readers do not. Also, it could be that good and poor readers respond differently to different types of feedback.

In the present study, both words and pseudowords were included, to see whether different reading routes (DRC) resulted in different effects. The study had a pretest, training, posttest, retention design that allowed us to look at direct and retention effects, and at the effects on trained and untrained items. We measured reading accuracy and speed on ten consecutive sessions within a time frame of 2 weeks, followed by a retention test 2 weeks later. Furthermore, transfer to untrained words and pseudowords was measured via a pre- and posttest directly before and after the training. The research questions were as follows:

1. Does immediate corrective feedback in repeated word reading, either on the word or phonics level, result in larger direct, retention, and transfer effects compared to repeated word reading without feedback?
2. What are the differences between good and poor readers with regard to direct, retention, and transfer effects?

Regarding the first research question, we expected that the inclusion of feedback (both word and phonics) would result in larger direct, retention, and transfer effects compared to the no feedback control condition. With regard to the second research question, we expected to find direct, retention, and transfer effects for both accuracy and speed for both groups of readers. The materials that we used are relatively simple CVC (consonant, vowel, consonant) items. We therefore expected that accuracy and speed for good readers of the first grade would already be very high at the start of the intervention. We thus expected that poor readers would show a stronger increase on accuracy and especially on speed than the good readers, because in this former group of readers, there is more room for improvement. Moreover, we expected phonics feedback, which stresses the grapheme-phoneme rules and thus the indirect route of reading, to be more effective in poor readers than in good readers, since we assumed that they swiftly use the direct route of reading for these simple items.

To answer our research questions we developed a repeated reading computer intervention. There was a training set of 50 (pseudo) words and a transfer set of 50 different (pseudo) words which had to be read out aloud by the participants. The training set was presented for ten consecutive times during a 2-week intervention and once again 2 weeks after the intervention to measure retention. The transfer set was presented prior to and directly following the intervention. Both good and poor readers were divided over three conditions: no feedback, feedback at the word level, and feedback at the phonics level.

## Method

### Participants

Participants were 95 monolingual Dutch first graders (51 boys, 44 girls) with a mean age of 6 years and 9 months (SD 4 months, range 73–93 months). Half of the participants (47) were poor readers. They were defined as poor readers in the present study based on a score below the 25th percentile on a standardized isolated word reading task (Three-Minute-Test, Verhoeven, 1995). The other half of the participants (48) was a good reading control group (i.e., above the 75th percentile). The scores on this word reading task represent word reading efficiency, a measure which includes both accuracy and speed. All participants were native speakers of Dutch.

The participating children came from eight different middle class schools located in the eastern and southern regions in the Netherlands. All schools used the same phonics-based reading and spelling method called “Veilig leren lezen” (Learning to read safely, Mommers, Verhoeven, & Van der Linden, 1990). We obtained written consents of the parents of the children, with assurance of anonymity.

The data of this study was collected in two different rounds, which have been combined in this paper. None of these data have been published before. In the first round, we compared phonics feedback to word feedback; assignment of these two conditions was random. Due to some technological issues we could not use the majority of data from the “phonics” condition. One year later, we selected children with the same amount of reading experience for participation in our experiment. Apart from rerunning subjects in the “phonics” condition, we also added a “no feedback” control condition. Again, assignment to one of the two conditions was random. For poor readers, the two experiments combined resulted in 22 participants in the phonics feedback condition, 10 participants in the word feedback condition, and 15 in the no feedback condition. For good readers, the two experiments combined resulted in 22 participants in the phonics feedback condition, 14 participants in the word feedback condition, and 12 in the no feedback condition.

### Materials

#### Pretest measures

To assess several aspects of reading, standardized measures of all participants were taken prior to the reading training. Children were assessed on phonological memory, naming speed, and receptive vocabulary knowledge. Descriptive statistics for both groups of readers in all three conditions are presented in Table 1. In both groups of readers, there were no significant differences between the three conditions for all three measures. The good readers were significantly better in pseudoword repetition, *F*(1,93) = 13.10, *p* < 0.001, and naming speed, *F*(1,93) = 19.66, *p* < 0.001. The difference between groups for receptive vocabulary was not significant, *F*(1,93) = 2.38, *p* = 0.13.

**Table 1:** Mean scores for pretest measures for good and poor reading children in the phonics, word, and no feedback condition

	Good readers			Poor readers		
	Phonics	Word	No	Phonics	Word	No
*N*	22	14	12	22	10	15
Pseudoword repetition [40]	31.10 (5.22)	28.86 (4.29)	32.92 (6.01)	27.50 (5.56)	26.90 (5.86)	26.93 (4.93)
Naming speed [120]	57.27 (11.41)	56.00 (10.18)	61.08 (10.82)	48.86 (7.83)	45.10 (8.01)	50.93 (11.17)
Passive vocabulary [96]	77.05 (5.77)	78.43 (6.93)	79.83 (4.73)	75.95 (5.03)	77.10 (8.53)	76.33 (4.92)

Standard deviations are within parenthesis, maximum scores are presented within brackets

##### Pseudoword repetition

This task served as a measure of phonological memory and was taken from the Standardized Screening Test for Children with Specific Language Impairment (Verhoeven, 2006; Cronbach’s alpha = 0.95). In this task, participants have to repeat the pseudoword after the experimenter. The experimenter reads out aloud 40 pseudowords consecutively, increasing in length and difficulty. The total number of correctly repeated items was scored.

##### Naming speed

This task served as a measure of lexical retrieval and was also taken from the Standardized Screening Test for Children with Specific Language Impairment (Verhoeven, 2006; Cronbach’s alpha = 0.95). We used the picture naming task in which participants are asked to name pictures in a serial naming task. The task consisted of five randomly occurring pictures. Children had to name as many pictures within 1 min. The total number of correctly named pictures was scored.

##### Receptive vocabulary

To measure vocabulary knowledge in the participating children, we administered the receptive vocabulary task from the Language Test for Children (Verhoeven & Vermeer, 2001; Cronbach’s alpha = 0.95). This task consists of 96 items and two practice items. Each item is constructed as follows. The experimenter reads out a word, and the participant has to choose between four pictures which picture illustrates the word read out. If five errors were made in a row, the test was terminated. Only correct answers were summed up and scored.

#### Stimuli

The stimuli were the same as in the study of Van Gorp et al. (2014). All stimuli were orthographically transparent CVC items. They were created with the use of 14 frequent used graphemes; five short vowels and nine consonants. From these graphemes, 100 items were created. Of those items 50 were CVC words selected from a database of words known by 6-year-olds (Schaerlaekens, Kohnstamn, Lejaegere, de Vries, Peeters & Zink, 1999). All words were high frequent nouns. The selection criteria of the words were that they consisted of three letters (belonging to the set of 14 graphemes) and that they were transparent. Also, the selected words were actively known by at least 70 % of Dutch 6-year-olds, according to Schaerlaekens et al. (1999). From these 50 words, 50 matching CVC pseudowords were created by scrambling the letters. From these 100 items, two lists labeled A and B were created (see Appendix).

#### Procedure

Prior to the intervention period, pretests were administered during a 30-min session. All repeated reading sessions lasted approximately 5–10 min. Words and pseudowords were offered blockwise. The order of the blocks was counterbalanced between participants and sessions. Items were pseudo-randomized within these blocks. Items were randomized in such a way that two subsequent items could have maximally one of three positions (CVC) in common in order to prevent orthographic neighborhood effects.

**Fig. 1:**
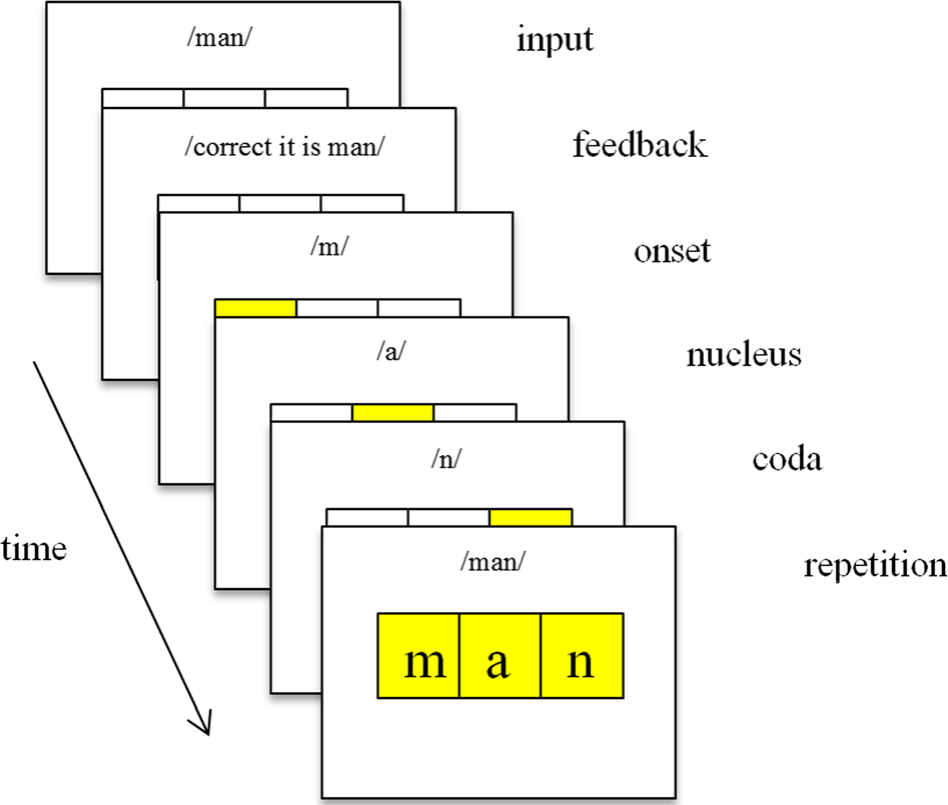
Schematic representation of procedure, as was used in Van Gorp et al. (2014). The input reflects the word as represented on the screen. The child reads the word out aloud and the experimenter indicates whether it was correct or not. For the children in the *no feedback condition* the experiment proceeded to the next item. For the children in the *word feedback condition* the second screen appeared, accompanied by auditory feedback stating what the correct representation should be. For the children in the *phonics feedback condition* the other four screens apply as well. After stating the correct representation, the item was spelled out and repeated once more, while graphemes and the word lit up accordingly

All repeated word reading sessions took place within the schools of the participating children, in a room separate from their classroom. We used a laptop for presentation of the items and a headphone/microphone for the auditory feedback and for the recordings of the items read by the participants. Items were presented as black letters in the middle of a white screen. Each letter was presented in a separate box, as illustrated in Fig. 1. Items remained visible on the screen for an unlimited duration. After the participants read the word, the experimenter indicated whether the item was read correctly or incorrectly and the experiment proceeded to the next item. Depending on the condition the participants were in, they received feedback as initiated by the experimenter’s judgment. By pressing button “1” on an external keyboard, the experimenter indicated that the item was read correctly; incorrect items were indicated by pressing button “2.”

For the children who were in one of the two feedback conditions, a voice-over character providing the feedback was introduced to them prior to the first reading session. In these conditions, children received feedback on both correct and incorrect read items. In the *word feedback condition*, the character told whether the word pronounced by the children was correct or not, followed by the pronunciation in the correct way, regardless of the correctness (i.e., correct, it is *cat*). For the *phonics feedback condition*, the feedback consisted of the word feedback plus the spelling out the sounds of the onset, nucleus, and coda, followed by spelling out the whole word (i.e., incorrect, it is *cat*, *k*-*æ*-*t*, *cat*). During this spelling out and repetition of the whole word, the boxes in which the letters were lit up corresponding with the spelling out of the letters. In the *no feedback condition*, there was no feedback at all. After reading out aloud an item, the experimenter pressed a button (i.e., correct or incorrect) and the experiment continued. For a schematic presentation of the feedback procedure, see Fig. 1. In the two conditions with feedback, all participants received feedback on all trials during all sessions, thus for both incorrect and correct responses. After the feedback (or directly after the response in the no feedback condition) a fixation cross appeared on the screen and the next word was presented.

The experimenter rated the answers of the children online by means of an external keyboard. By pressing the key on the keyboard, latencies were measured as well. The experiment was programmed in E-prime software, Version 2.0 (Psychology Software Tools, Pittsburgh, PA). Each item was individually recorded via the microphone and recorder function in E-prime. For backup purposes, all sessions were recorded on an external voice recorder as well. Since rating speed of the four different experimenters varied quite a lot, all items were re-assessed by means of Praat (Boersma, 2001), resulting in more reliable data. Each individual item was analyzed semi-automatically by Praat resulting in a precise onset-latency (when the participant started pronouncing the item) and offset-latency (when the item was fully pronounced).

#### Data analysis

For all items, accuracy, onset-latency, and offset-latency were measured. In the analyses concerning reading latencies, only correct responses were included. All items with an onset-latency higher than 5000 ms were removed (4.97 %). In addition, incorrect responses were removed (another 4.14 %). For each child and each session, mean reading latencies and mean accuracy scores for words and pseudowords were calculated. If a participant did not participate during one of the sessions, this participant was removed from analysis of that measurement (direct, retention, transfer). For direct and retention effects, two good readers (both phonics condition) and one poor reader (phonics condition) were removed from analysis. For transfer effects, one good reader (word condition) and one poor reader (no feedback condition) were removed from analysis.

In studies using voice keys, the onset time is usually reported, but we chose to report the offset times, because it is likely to be a more reliable measure for reading latencies in children, since it includes naming duration as well (Huemer et al., 2010; De Jong & Share, 2007; Thaler, Ebner, Wimmer, & Landerl, 2004). To examine change over time, repeated measures ANOVAs were conducted. If the assumption of sphericity was not met for the main effect of time, degrees of freedom were corrected using Greenhouse-Geisser estimates of sphericity.

Our two research questions address the effects of various types of feedback and differences between good and poor readers. These were examined in combined analyses: repeated measures analyses with time and word type as within-subject variables and condition and reading level as between-subject variables. These analyses were conducted to assess direct effects, retention effects, and transfer effects. Each of these three analyses were performed for both reading accuracy (i.e., percentage correct responses) and for reading speed.

## Results

In Tables 2 and 3, mean reading latencies for all measurement points for all three conditions are represented for poor and good readers, respectively. For speed, we only used the correct responses.

**Table 2:** Mean offset reading accuracy (in percentage correct) and latencies (in milliseconds) for all three conditions for each measurement point for the poor readers

		Pretest	T1	T2	T3	T4	T5	T6	T7	T8	T9	T10	Posttest	Retention
Phonics feedback (N = 22)	Words accuracy	0.95 (0.06)	0.94 (0.06)	0.93 (0.08)	0.94 (0.06)	0.95 (0.04)	0.95 (0.05)	0.96 (0.05)	0.94 (0.06)	0.94 (0.06)	0.93 (0.10)	0.96 (0.06)	0.93 (0.07)	0.95 (0.06)
	Words latency	2885 (464)	2871 (708)	2689 (643)	2677 (614)	2624 (616)	2677 (585)	2712 (547)	2579 (602)	2515 (586)	2614 (567)	2642 (521)	2886 (560)	2644 (599)
	Pseudowords accuracy	0.92 (0.07)	0.09 (0.09)	0.92 (0.08)	0.92 (0.09)	0.93 (0.07)	0.92 (0.07)	0.92 (0.07)	0.92 (0.07)	0.94 (0.06)	0.93 (0.06)	0.93 (0.07)	0.91 (0.07)	0.93 (0.07)
	Pseudowords latency	3302 (606)	2871 (671)	2964 (665)	2983 (633)	2913 (529)	2922 (648)	2904 (544)	2834 (532)	2733 (612)	2784 (553)	2787 (592)	2971 (632)	2768 (657)
Word feedback (*N* = 10)	Words accuracy	0.92 (0.08)	0.90 (0.12)	0.94 (0.08)	0.86 (0.14)	0.89 (0.10)	0.89 (0.13)	0.90 (0.09)	0.92 (0.08)	0.86 (0.15)	0.85 (0.14)	0.92 (0.08)	0.87 (0.11)	0.92 (0.05)
	Words latency	3208 (487)	3172 (603)	3017 (610)	2967 (883)	2901 (681)	3040 (798)	2764 (773)	2961 (689)	2753 (595)	2743 (635)	2770 (571)	3166 (710)	2837 (528)
	Pseudowords accuracy	0.85 (0.09)	0.85 (0.14)	0.87 (0.07)	0.87 (0.08)	0.85 (0.14)	0.83 (0.18)	0.81 (0.18)	0.88 (0.13)	0.88 (0.10)	0.80 (0.17)	0.88 (0.15)	0.84 (0.17)	0.87 (0.10)
	Pseudowords latency	3676 (232)	3172 (587)	3291 (608)	3211 (736)	3096 (803)	3241 (853)	3275 (740)	3165 (658)	3176 (676)	3117 (790)	3141 (711)	3279 (765)	2945 (575)
No feedback (*N* = 15)	Words accuracy	0.95 (0.06)	0.93 (0.07)	0.91 (0.11)	0.93 (0.06)	0.91 (0.07)	0.94 (0.06)	0.95 (0.05)	0.95 (0.07)	0.94 (0.05)	0.92 (0.15)	0.96 (0.03)	0.95 (0.06)	0.96 (0.04)
	Words latency	2515 (570)	2535 (658)	2584 (527)	2461 (613)	2289 (570)	2345 (621)	2357 (563)	2224 (537)	2325 (628)	2402 (537)	2299 (528)	2668 (579)	2205 (591)
	Pseudowords accuracy	0.92 (0.08)	0.90 (0.09)	0.89 (0.09)	0.91 (0.06)	0.90 (0.08)	0.98 (0.07)	0.92 (0.04)	0.92 (0.07)	0.94 (0.07)	0.91 (0.04)	0.94 (0.07)	0.92 (0.08)	0.95 (0.06)
	Pseudowords latency	2910 (613)	2865 (746)	2806 (693)	2748 (642)	2616 (626)	2709 (632)	2726 (517)	2645 (633)	2713 (677)	2705 (618)	2641 (545)	2907 (572)	2561 (634)

Standard deviations are between parentheses

**Table 3:** Mean offset reading accuracy (in percentage correct) and latencies (in milliseconds) for all three conditions for each measurement point for the good readers

		Pretest	T1	T2	T3	T4	T5	T6	T7	T8	T9	T10	Posttest	Retention
Phonics feedback (*N* = 22)	Words accuracy	0.99 (0.02)	0.99 (0.02)	0.99 (0.01)	1.00 (0.01)	1.00 (0.00)	1.00 (0.01)	1.00 (0.01)	1.00 (0.01)	0.99 (0.01)	1.00 (0.01)	0.99 (0.02)	0.99 (0.02)	1.00 (0.01)
	Words latency	1390 (304)	1335 (200)	1322 (213)	1375 (318)	1346 (235)	1306 (218)	1358 (299)	1392 (272)	1286 (213)	1294 (209)	1332 (289)	1383 (320)	1278 (243)
	Pseudowords accuracy	0.99 (0.02)	0.98 (0.02)	0.99 (0.02)	0.98 (0.03)	0.99 (0.03)	0.99 (0.03)	0.99 (0.02)	0.99 (0.03)	0.99 (0.02)	0.99 (0.02)	0.99 (0.01)	0.99 (0.02)	1.00 (0.01)
	Pseudowords latency	1595 (358)	1462 (236)	1426 (264)	1405 (304)	1405 (296)	1329 (255)	1363 (219)	1347 (267)	1319 (199)	1322 (263)	1360 (292)	1465 (339)	1285 (236)
Word feedback (*N* = 13)	Words accuracy	0.99 (0.03)	0.98 (0.03)	0.99 (0.01)	1.00 (0.01)	0.99 (0.05)	0.99 (0.02)	0.99 (0.02)	0.99 (0.01)	0.99 (0.01)	0.99 (0.01)	0.99 (0.02)	1.00 (0.01)	1.00 (0.00)
	Words latency	1476 (269)	1522 (349)	1544 (497)	1507 (455)	1622 (438)	1421 (255)	1458 (317)	1502 (260)	1476 (324)	1424 (261)	1520 (293)	1620 (404)	1477 (297)
	Pseudowords accuracy	0.98 (0.03)	0.99 (0.02)	0.98 (0.03)	0.97 (0.03)	0.99 (0.02)	0.98 (0.03)	0.97 (0.03)	0.99 (0.02)	0.99 (0.02)	0.99 (0.02)	0.99 (0.01)	0.99 (0.03)	0.99 (0.02)
	Pseudowords latency	1833 (487)	1739 (339)	1764 (584)	1589 (440)	1630 (329)	1589 (427)	1608 (351)	1632 (309)	1565 (307)	1448 (227)	1516 (317)	1707 (354)	1493 (201)
No feedback (*N* = 12)	Words accuracy	1.00 (0.01)	1.00 (0.01)	1.00 (0.00)	1.00 (0.00)	1.00 (0.01)	1.00 (0.01)	1.00 (0.01)	1.00 (0.00)	1.00 (0.01)	1.00 (0.01)	1.00 (0.00)	0.99 (0.02)	0.99 (0.02)
	Words latency	1216 (159)	1236 (249)	1194 (160)	1252 (250)	1170 (196)	1146 (248)	1189 (179)	1210 (238)	1133 (194)	1122 (244)	1155 (247)	1214 (200)	1140 (211)
	Pseudowords accuracy	0.99 (0.02)	1.00 (0.01)	0.99 (0.02)	0.99 (0.02)	1.00 (0.01)	0.99 (0.02)	0.99 (0.02)	1.00 (0.00)	1.00 (0.00)	0.99 (0.02)	1.00 (0.00)	0.99 (0.02)	1.00 (0.01)
	Pseudowords latency	1330 (189)	1262 (212)	1246 (162)	1223 (176)	1255 (178)	1205 (178)	1235 (232)	1213 (208)	1238 (215)	1176 (246)	1161 (241)	1253 (202)	1208 (276)

Standard deviations are between parentheses

### Accuracy

#### Direct effects of the training on accuracy

The first research question addressed the effects of various types of feedback, the second differences between good and poor readers. Both questions were first examined with regard to improvement of accuracy on direct effects in one analysis. An ANOVA of repeated measures with time (T1, T2, …, T10) and word type (words, pseudowords) as within-subjects factors, and condition (no feedback, phonics feedback, word feedback) and reading level (poor readers, good readers) as between-subjects factors was performed on the mean reading accuracy.

All significant main and interaction effects are reported in Table 4. The main effect for time was further examined with planned comparisons (repeated), but between subsequent sessions, no significant differences were found. The main effect thus reflects of an overall growth of reading accuracy. To answer our first research question, we were interested in interactions including both condition and time. These interactions were not found, suggesting that there were no differences in growth between conditions.

**Table 4:** Main and interaction effects of intervention measure from repeated measures ANOVA with time and word type (within) and condition and reading level (between) as factors

	*df*	*F*	*p*	η^2^_p_
Time				
Accuracy	6.42, 551.72	2.40	0.02	0.03
Speed	5.11, 439.42	7.74	<0.001	0.08
Word type				
Accuracy	1, 86	41.20	<0.001	0.32
Speed	1, 86	167.92	<0.001	0.66
Condition				
Accuracy	2, 86	6.04	<0.01	0.12
Speed	2, 86	7.11	<0.01	0.14
Reading level				
Accuracy	1, 86	95.49	<0.001	0.53
Speed	1, 86	259.97	<0.001	0.75
Time × reading level				
Accuracy	6.42, 551.72	1.49	NS	
Speed	5.11, 439.42	2.84	0.02	0.03
Word type × reading Level				
Accuracy	1, 86	19.99	<0.001	0.19
Speed	1, 86	72.12	<0.001	0.46
Condition × reading level				
Accuracy	2, 86	4.14	0.02	0.09
Speed	2, 86	0.27	NS	

*NS* not significant

Regarding our second research question, we were interested in interactions with reading level. For accuracy we only found and interaction of condition × reading level. To quantify this interaction, separate analyses for poor and good readers were performed. For good readers, there was a medium-sized effect of condition, *F*(2, 43) = 3.99, *p* = 0.03, η^2^_p_ = 0.16. Bonferroni post hoc analysis showed that good readers have a higher accuracy score at the *no feedback* condition than at the *word feedback* condition (*p* = 0.02). Between the *phonics feedback* condition and the *word feedback* condition, there was no difference (*p* = 0.20), neither was there a difference between the *phonics feedback* condition and the *no feedback* condition (*p* = 0.72). For poor readers, there also was a medium-sized effect of condition, *F*(2, 43) = 4.61, *p* = 0.02, η^2^_p_ = 0.18. Bonferroni post hoc analysis showed that poor readers had higher accuracy scores at the *phonics feedback* condition than at the *word feedback* condition (*p* = 0.01). There was no difference between *no feedback* and *phonics feedback* (*p* = 1.00) and the difference between *no feedback* and *word feedback* was marginally significant (*p* = 0.08). There were thus differences between good and poor readers with regard to direct effects on accuracy in the type of feedback that they received. Good readers showed the highest accuracy scores in the *no feedback* condition, whereas poor readers benefitted most of the *phonics feedback* condition.

#### Retention effects of the training on accuracy

Next, we examined both research questions with regard to retention of accuracy. To measure if the increased accuracy level was retained 2 weeks after training, an ANOVA of repeated measures with time (T1, T10, retention) and word type (words, pseudowords) as within-subjects factors and condition (no feedback, phonics feedback, word feedback) and reading level (poor readers, good readers) as between-subjects factors was performed on the mean reading accuracy.

All significant main and interaction effects are reported in Table 5. To answer our research questions we were mainly interested in interaction effects of Condition or Reading Level with Time, but for retention of accuracy we found none of these interactions to be significant. To examine whether there was a retention effect, we further examined the main effect of Time. Planned contrasts (Helmert) revealed that the effect of time between T1 and later was small but significant, *F*(1,86) = 10.86, *p* = 0.001, η^2^_p_ = 0.11. The difference between T10 and retention was not significant (*F* < 1), indicating that the reached level at T10 was retained two weeks later. With regard to the first research question, we can conclude that there were no differential effects of feedback on the accuracy of the retention measure. With regard to the second research question, we can conclude that there were no different effects between good and poor readers.

**Table 5:** Main and interaction effects of retention measure from repeated measures ANOVA with time and word type (within) and condition and reading level (between) as factors

	*df*	*F*	*p*	η^2^_p_
Time				
Accuracy	1.86, 160.07	6.50	<0.01	0.07
Speed	1.62,139.20	24.39	<0.001	0.22
Word type				
Accuracy	1, 86	14.23	<0.001	0.14
Speed	1, 86	69.71	<0.001	0.45
Condition				
Accuracy	2, 86	4.41	0.02	0.09
Speed	2, 86	8.04	<0.01	0.16
Reading level				
Accuracy	1, 86	79.75	<0.001	0.48
Speed	1, 86	277.22	<0.001	0.76
Time × reading level				
Accuracy	?	?	NS	
Speed	1.62, 139.20	7.59	<0.01	0.08
Word type × reading level				
Accuracy	1, 86	14.53	<0.001	0.15
Speed	1, 86	30.48	<0.001	0.26

*NS* not significant

#### Transfer effects of the training on accuracy

Third, to assess whether the intervention had any transfer effects for accuracy, an ANOVA of repeated measures with Time (transfer1, transfer2) and Word Type (words, pseudowords) as within-subjects factors and Condition (no feedback, phonics feedback, word feedback) and Reading Level (poor readers, good readers) as between-subjects factors was performed on the mean reading accuracy.

All significant main and interaction effects are reported in Table 6. There was no main effect for time (*F*(1,87) = 1.90, *p* = 0.17, = η^2^_p_ = 0.02) indicating that accuracy did not improve for untrained items. Note that overall accuracy was already above 90 % for the poor readers and above 98 % for the good readers at transfer1, indicating that there was little room for improvement.

**Table 6:** Main and interaction effects of transfer measure from repeated measures ANOVA with time and word type (within) and condition and reading level (between) as factors

	*df*	*F*	*p*	η^2^_p_
Time				
Accuracy	1, 87	1.90	NS	
Speed	1, 87	3.64	0.06	0.04
Word Type				
Accuracy	1, 87	25.19	<0.001	0.23
Speed	1, 87	81.73	<0.001	0.48
Condition				
Accuracy	2, 87	4.28	0.02	0.09
Speed	2, 87	9.43	<0.001	0.18
Reading Level				
Accuracy	1, 87	73.68	<0.001	0.46
Speed	1, 87	358.43	<0.001	0.81
Time × reading level				
Accuracy	1, 87	2.76	0.10	0.03
Speed	1, 87	1.00	NS	
Word type × reading level				
Accuracy	1, 87	17.02	<0.001	0.16
Speed	1, 87	7.98	<0.01	0.08
Condition × reading level				
Accuracy	2, 87	3.67	0.03	0.08
Speed	2, 87	0.22	NS	

*NS* not significant

To answer our first research question, we were particularly interested in interaction effects including both condition and time. None of these interactions were found. For our second research question, we were interested in interactions with reading level. We found an interaction of condition × reading level. Moreover, we found an interaction of word type × reading level. The interaction of time × reading level was approaching significance. To quantify these two-way interactions with reading level, we performed a repeated measures ANOVA with time, word type and condition for good and poor readers separately. For the good readers, no effect for condition (*F* < 1) was found. There were also no effects for time (*F* < 1) or Word Type (*F* < 2). For the poor readers there was a medium sized significant effect of condition, *F*(2,43) = 3.76, *p* = 0.03, η^2^_p_ = 0.15. Bonferroni post hoc comparisons revealed that there was no difference between *phonics feedback* and *no feedback* for the group of poor readers (*p* = 1.00). But both *phonics feedback* and *no feedback* resulted in higher accuracy scores than *word feedback* (both pairs: *p* = 0.05). For the poor readers there was also no main effect for Time (*F* < 3). There was a large main effect for Word Type (*F*(1,43) = 22.62, *p* < 0.001, η^2^_p_ = 0.35) indicating that words were read more accurately than pseudowords in poor readers. With regard to the first research question, we did not find any differential effects of feedback on the accuracy of the transfer task. With regard to the second research question, we did find differences between good and poor readers. In poor readers there was an effect for condition, favoring phonics and word feedback over no feedback, whereas in good readers, there was no effect of feedback on accuracy of transfer.

#### Speed

We next performed the same set of analyses as for the accuracy data, but this time for direct effects, retention effects, and transfer effects on reading speed. Again comparing effects of various types of feedback (question 1) and differences between good and poor readers (question 2).

#### Direct effects of the training on speed

To assess direct effects of reading speed, an ANOVA of repeated measures with time (T1, T2, …, T10) and word type (words, pseudowords) as within-subjects factors and condition (no feedback, phonics feedback, word feedback) and reading level (poor readers, good readers) as between-subjects factors was performed on the mean offset reading latencies.

All main and interaction effects are reported in Table 4. To answer the first research question we were interested in interactions including both condition and time. These interactions were not found. To answer the second research question, we were particularly interested in interactions with reading level. For speed, we found a small but significant interaction effect of time × reading level, indicating that poor readers increased more over time than good readers.

To quantify this interaction effect and to be able to quantify the differences between good and poor readers we performed the repeated measures analysis again on poor and good readers separately. Because we also found an interaction of word type × reading level, we also performed analyses for words and pseudowords separately. In these analyses, condition was not included, since post hoc tests revealed no differences between the three conditions. Four separate repeated measures ANOVA with time (T1 to T10) as within-subject factor were performed. Planned contrasts were used to test at which sessions speed increased and at which sessions speed remained stable. Repeated contrasts were used to see which sessions differed from its subsequent session and Helmert contrasts to see how each session differed from all following sessions combined.

##### Direct effects of the training on speed for the good readers

**Fig. 2:**
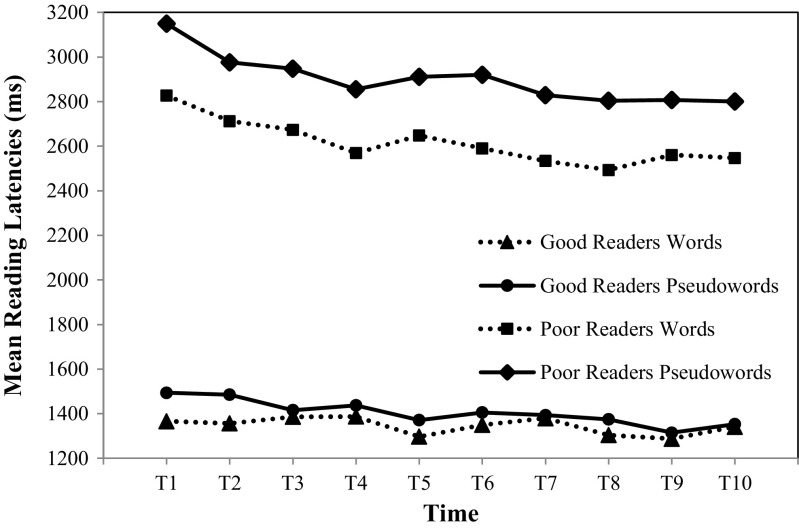
Mean reading latencies for the good and poor readers. Scores represent overall mean reading latencies in milliseconds for each consecutive measurement. The *dotted line* represents reading latencies for words, the *solid line* represents reading latencies for pseudowords

For the good readers we found no effect of Time for words, *F*(3.85, 173.18) = 1.93, *p* = 0.11, η^2^_p_ = 0.04. As visualized in Fig. 2 it can be assumed that there was too much variation in reading times to find an effect of time. For this group of readers a small effect of Time for pseudowords was found, *F*(4.91, 220.87) = 4.71, *p* < 0.001, η^2^_p_ = 0.10. Repeated contrasts reveal that there are only small significant increases in reading speed from T2 to T3, *F*(1,45) = 4.20, *p* = 0.05, η^2^_p_ = 0.09, from T4 to T5, *F*(1,45) = 4.85, *p* = 0.03, η^2^_p_ = 0.10 and from T8 to T9, *F*(1,45) = 4.93, *p* = 0.03, η^2^_p_ = 0.10. This progress can be seen in Fig. 2. The difference between the current session and subsequent sessions is only significant with effects of medium size as shown by Helmert contrasts for T1, *F*(1,45) = 13.90, *p* = 0.001, η^2^_p_ = 0.24, T2, *F*(1,45) = 6.48, *p* = 0.01, η^2^_p_ = 0.13 and T4, *F*(1,45) = 8.80, *p* = 0.01, η^2^_p_ = 0.16.

##### Direct effects of the training on speed for the poor readers

For the poor readers we found a small effect of time for words, *F*(6.25, 281.01) = 4.18, *p* < 0.001, η^2^_p_ = 0.09. See Fig. 2 for a graph of the mean reading latencies for this group of readers. Repeated contrasts revealed a small significant increase in reading speed from T1 to T2, *F*(1,45) = 4.44, *p* = 0.04, η^2^_p_ = 0.09 and a marginally significant increase in speed from T3 to T4, *F*(1,45) = 3.55, *p* = 0.07, η^2^_p_ = 0.07. As visualized in Fig. 2, an asymptote for words is reached at T4. This is confirmed by Helmert contrasts which showed that the difference between T1 and later is significant, *F*(1,45) = 17.38, *p* < 0.001, η^2^_p_ = 0.28, which can be classified as a large effect. The difference between T2 and later, *F*(1,45) = 6.37, *p* = 0.02, η^2^_p_ = 0.12 and the difference between T3 and later, *F*(1,45) = 4.21, *p* = 0.05, η^2^_p_ = 0.09 were small but significant. For pseudowords we also found a small effect of time in the group of poor readers, *F*(6.15, 276.53) = 4.90, *p* < 0.001, η^2^_p_ = 0.10. Here, there only was a medium-sized significant increase in reading speed from T1 to T2 (see Fig. 2), *F*(1,45) = 7.13, *p* = 0.01, η^2^_p_ = 0.14. As depicted in Fig. 2 an asymptote for pseudowords is also reached at T4. This was confirmed by Helmert Contrasts that show that the difference between T1 and later is significant, *F*(1,45) = 18.44, *p* < 0.001, η^2^_p_ = 0.29, which can be classified as a large effect. The difference between T2 and later, *F*(1,45) = 5.17, *p* = 0.03, η^2^_p_ = 0.10 and the difference between T3 and later, *F*(1,45) = 3.87, *p* = 0.06, η^2^_p_ = 0.08 were small but significant.

With regard to the first research question, it can be concluded that there were no differences between the different types of feedback on the direct effects on speed. With regard to the second research question, we did find differences between good and poor readers. Good readers seem to only progress significantly on pseudowords over time. Poor readers improved their speed in both words and pseudowords. The asymptote for those effects seems to be equal for good and poor readers and was reached around T4.

#### Retention effects of the training on speed

To see if the trained speed was retained 2 weeks later, we performed a repeated measures ANOVA with time (T1, T10 and retention) and word type (words, pseudowords) as within-subjects factors and condition (no feedback, phonics feedback, word feedback) and reading level (poor readers, good readers) as between-subjects factors was performed on the mean offset reading latencies.

All significant main and interaction effects are presented in Table 5. To be able to find out if reading speed was retained, planned comparisons were made. Helmert comparisons revealed differences between T1 and the two later measurements (T10 and retention), *F*(1,86) = 32.09, *p* < 0.001, η^2^_p_ = 0.27, indicating a large effect for growth in reading speed from T1 to the measurements T10 and Retention. The difference between T10 and Retention was not significant, *F*(1,86) = 2.19, *p* = 0.14, η^2^_p_ = 0.03, indicating that the average reading speed at retention was similar to the average reading speed at T10. This means that the trained speed was retained 2 weeks later.

To answer our first research question, it is important to acknowledge that there were no interaction effects for condition. To be able to answer the second research question, we were interested in interactions with reading Llvel. The interaction of time × reading level shows that poor readers improve more over time than good readers. The interaction effect of word type × reading level showed that the difference between words and pseudowords is larger for poor readers than for good readers. Thus, both good and poor readers retained their increased speed levels. The growth for poor readers, however, was larger than for good readers. Moreover, the poor readers showed larger differences between words and pseudowords than the good readers.

#### Transfer effects of the training on speed

To see if there were transfer effects for speed, an ANOVA of repeated measures with time (transfer1, transfer2) and word type (words, pseudowords) as within-subjects factors and condition (no feedback, phonics feedback, word feedback) and reading level (poor readers, good readers) as between-subjects factors was performed on the mean offset reading latencies.

All significant main and interaction effects are reported in Table 6. For the main effect of Condition, Bonferroni post hoc analysis revealed that children in the *no feedback* condition are faster than the children in the *word feedback* condition (*p* = 0.02). The difference between *phonics feedback* and *word feedback* (*p* = 0.36) and the difference between *phonics feedback* and *no feedback* (*p* = 0.30) was not significant.

To answer the question whether we found a transfer effect here, rather than orthographic learning or a testing effect we performed another analysis. An ANOVA of repeated measures with item list (transfer, training), time (measurement1, measurement2) and word type (words, pseudowords) as within-subjects factors and condition (no feedback, phonics feedback, word feedback) and reading level (poor readers, good readers) as between-subjects factors was performed on the mean offset reading latencies. If transfer indeed occurred, we would find an interaction of item list × time, indicating that the increase from measurement 1 to measurement 2 for the trained items differed from the increase for transfer items.

The two-way interaction effect for item list × Time was not significant (*F* < 1), however, there was a small significant three-way interaction effect for item list × Time × word type, *F*(1.84) = 7.82, *p* < 0.01, η^2^_p_ = 0.09. Follow up analyses revealed that there was an marginally significant interaction for item list × time for the pseudowords, *F*(1.84) = 3.34, *p* = 0.07, η^2^_p_ = 0.04, and an even smaller effect for the words, *F*(1,84) = 2.70, *p* = 0.10, η^2^_p_ = 0.03. The directions of these effects, however, were in opposite direction of each other. For pseudowords, the effect of the transfer task was larger from measurement 1 to measurement 2, whereas for the words the direct effect was larger. All these data show us that we did find transfer effects for pseudowords, but no transfer effects for words.

To answer our first research question we were interested in interaction effects including condition and time. These effects were not found, indicating that there were no differences between the different types of feedback. To answer our second research question, we were interested in interaction effects with Reading Level. The only significant interaction was the one of reading level × word type which indicates that the difference between words and pseudowords was larger for poor readers than for good readers. This implies that the differences between good and poor readers with regard to transfer effects of speed were small. Moreover, there was evidence of transfer of pseudowords for both types of readers.

## Discussion

The main interest of this study was to investigate the effects of repeated word reading on good compared to poor readers in first grade, and the influence of feedback on these effects. In the past, repeated reading has turned out to be an effective method to increase word reading fluency in trained words (e.g., Berends & Reitsma, 2006; Lemoine et al.,^1993^). Transfer to untrained words, however, has only been found in a limited amount of studies (e.g., Thaler et al., 2004; Van Gorp et al., 2014). Based on these two previous studies, we assumed that the inclusion of corrective feedback might induce transfer effects. Therefore, we compared repeated word reading in two feedback conditions (phonics feedback and word feedback) to a condition without feedback. For accuracy, we found that for the direct training effects, good reading children were most accurate in the no feedback condition, whereas poor reading children were the most accurate in the phonics feedback condition. But in both cases, this effect was only present as compared to the word feedback condition. There was no influence of feedback for speed.

Our second research question was whether there were differences between good and poor readers with regard to repeated word reading in terms of direct, retention and transfer effects. For accuracy, we mainly found results that indicated that good readers benefitted from the no feedback condition, whereas poor readers performed more accurately in the phonics feedback condition. For speed, we found similar results for good and poor readers. However, poor readers turned out to increase more over time. Transfer effects were found for speed for pseudowords for both types of readers.

### Feedback does not influence the effect of repeated reading

The first research question regarded the influence of various types of feedback compared to no feedback on the effect of repeated word reading. We assumed that the inclusion of feedback would result in finding transfer effects, following the common factor in the studies of Huemer et al. (2010) (on syllable reading), Van Gorp et al. (2014) (on word reading), and Young, Bowers and MacKinnon (1996) (on text reading). We also predicted that phonics feedback would be particularly helpful for poor readers, since this would be stressing the indirect route of reading. It turned out that type of feedback had a minimal effect on the direct effect on accuracy. At the transfer task, for accuracy we found a small interaction effect of condition × reading level. For good readers, there was no effect of condition. For poor readers, it was found that children from the phonics feedback condition and the no feedback condition outperformed children in the word feedback condition. There was no significant difference between the phonics feedback condition and the no feedback condition. For speed, there was no interaction of condition. It thus turns out that the inclusion of feedback in a repeated word reading intervention does not necessarily help the beginning reader. Our assumption that the inclusion of feedback caused transfer effects to occur was thus not confirmed. And even though there was a minimal effect of phonics feedback in poor readers, this was only the case when compared to word feedback. Based on this study, we thus cannot conclude that phonics feedback is in particular effective for poor readers. A reason for not finding beneficial effects of feedback might be the need of the participants for corrective feedback. Corrective feedback is usually aimed at correcting something. In this case, accuracy was already at a very high level at the start of the intervention, indicating that feedback might not be very useful. Since good readers actually performed most accurate in the no feedback condition, feedback may have been disturbing their reading. This is in line with Hattie and Timperley (2007) who suggested that feedback can be seen as disturbing while building fluency. In the study of Huemer et al. (2010), feedback was given only at incorrect trials, which is likely to be less disturbing. In the study of Van Gorp et al. (2014), inexperienced readers were assessed, and for them, the feedback might have had an instructional function as well.

### Similarities and differences between good and poor readers

Our second research question was whether there would be differences between good and poor readers with regard to repeated reading effects. As expected, we found main effects of time on both accuracy and speed for the direct and the retention measure, indicating an overall increase of reading efficiency for both types of readers. These direct effects of repeated word reading are in line with previous research on repeated word reading (e.g., Berends & Reitsma, 2006; Huemer et al., 2010; Martens & De Jong, 2008; Thaler et al., 2004; Van Gorp et al., 2014). The beginning readers did not fall back 2 weeks after the intervention, which indicates that orthographic information has been stored (Hogaboam & Perfetti, 1978; Share, 2004). While measuring direct effects of speed, we found an asymptote after only four repetitions; reading speed did not increase significantly further. Reading the same items for ten times can thus be seen as overlearning, and perhaps this overlearning of words is effective (Lemoine et al., 1993). However, it should be noted that we did not assess this effect on the long run, and furthermore, the retention of the transfer effects was not assessed. Moreover, analyses of the direct effect revealed that speed of words was not improved in good readers, while it did improve in the poor readers. A plausible explanation for this finding is that these words were already automatized in the good readers. This is a pattern that was also observed in the transfer effects.

The transfer effects were of particular interest, because these effects would indicate that the children improved their general word decoding skills, rather than the improvement of reading of a particular set of words. Transfer effects to untrained items were only found for pseudowords, both in poor and good readers. Here we did not find any differences between good and poor readers, again. According to the dual route model of reading (Coltheart et al., 2001) pseudowords are read via the indirect route. When reading novel words, children and adults also make use of this indirect route of reading. In this indirect route, words are read letter by letter. Our results indicate that repeated word reading facilitates this beginning stage of reading. Pseudoword reading can be seen as a pure measure of decoding skill (Gough & Tunmer, 1986). This means that repeated reading of single words leads to improvement of word decoding skill. An explanation for the fact that we did not establish transfer effects for words, might be the nature of the used items. All items were very high frequent CVC words (e.g., *cat*). It is likely that first graders have encountered these items before. As suggested by the instance theory (Logan, 1988) the largest progress towards an orthographic representation is made directly after the first encounter. Having encountered these words before could also imply that children already read these words via the direct route. This prior orthographic knowledge of the transfer words could also explain the discrepancy between the present transfer effects and those found in our previous study (Van Gorp et al., 2014) with kindergartners. In these children, with no prior reading experience, both words and pseudowords were processed as novel words via the indirect route, similar as pseudowords for the participants in the present study who had more prior reading experience. In the direct effects, we found an increase via the indirect route for pseudowords and via the direct route for words. The stronger increase in pseudowords and thus the stronger effect on decoding also reflects in the direct effects for the good readers. Based on these findings, we can conclude that both good and poor readers benefitted from the training. Transfer effects, however, were only established for pseudowords in both types of readers.

### Poor readers benefit more from repeated reading than good readers

Our final expectation was that poor readers would show larger direct effects than good readers. For speed, we indeed found larger direct effects in poor readers than in good readers. The difference between the good and the poor readers became smaller, indicating that the poor readers benefitted more from the training. A similar interaction was found for speed for the retention task, mainly driven by the direct effect. That poor readers benefitted more from training, can be explained by the fact that orthographic learning is supposed to occur quickly, after the first few occasions (Logan, 1988; Share, 2004). After that, an asymptote is reached (Hogaboam & Perfetti, 1978; Reitsma, 1983), even though children still increase in reading speed over time (Verhoeven & Van Leeuwe, 2009). Our data support both these views; i.e., the largest increase is made from T1 to T2 and an asymptote is reached after four repetitions. The overall largest increase was found from T1 to T2 in poor readers for pseudowords; words that they never encountered before (see also Fig. 2). This orthographic learning pattern may explain why poor readers were able to catch up with the good readers to some extent, as the good readers may already have reached their asymptote at the beginning of the intervention for words, and not for pseudowords. We must note, however, that a large gap in reading speed between the two groups remained, for both type of words, indicating that a more elaborate intervention may be necessary for the poor readers (cf. Saine, Lerkkanen, Ahonen, Tolvanen, & Lyytinen, 2011). For accuracy we found no differences between the different groups of readers over time, which is probably due to the high accuracy scores at the start of the intervention. The hypothesis that poor readers benefit more from repeated word reading could thus be confirmed for speed only; poor readers benefit more from repeated word reading than good readers.

### Future directions and limitations

It was assumed that the inclusion of feedback in the studies by Huemer et al. (2010) and Van Gorp et al. (2014) was the reason for transfer to occur. In the present study only transfer to pseudowords was found, regardless of type of feedback or even inclusion of feedback. As mentioned above, it could be that in the study of Van Gorp et al. (2014) transfer effects for both words and pseudowords are found because of their (lack of) reading experience. We assume that repeated reading of words and pseudowords enhances decoding skills and inexperienced readers use decoding via the indirect route for both words and pseudowords. This could have caused the transfer effects in the study of Huemer et al. (2010) as well. In their study children practiced with syllables, and the transfer to multisyllabic pseudowords containing those syllables was measured. In the study of Huemer et al. (2010), it is thus likely that their participants only used the indirect route of reading as well during the transfer task. Taken these findings together, it might thus be that the effect of repeated reading is only transferring to untrained words when these words are still read via the indirect route of reading. Future research should investigate whether repeated reading of words and or pseudowords indeed only enhances decoding skills, rather than fluent reading skills. This can perhaps be done by including more complex words in the transfer task (compare: Huemer et al., 2010). By investigating more complex words, it is more likely that children rely on the indirect route of word reading, which is assumed to benefit from repeated reading.

The present study was successful in improving decoding skills in good and poor reading children of Dutch, an orthographically transparent language. However, there were two limitations that lead to suggestions for future research. The first is that we did not monitor motivation, whereas motivation is likely to have influenced the results (Rasinski, 1990). Repeated reading of 50 CVC items for 13 sessions might be boring for these young readers, especially when receiving corrective feedback on each item. Future research in this topic should consider ways to increase the motivation of these children during repeated word reading. Thaler et al. (2004) solved this by ending the training for those who reached a certain threshold. Another way to increase motivation, or at lease decrease the chance of boredom or weariness, is to provide feedback only on incorrect trials, as was done in the study by Huemer et al. (2010).

Children who are intrinsically motivated to read seek for challenge (Wigfield & Guthrie, 1997). The second limitation of this study follows up on the motivation aspect and lies in the amount of challenge that was offered to the participants. Repeatedly reading CVC words can be quite boring, especially if you are a good reader. A suggestion for future research is to include more challenge during training. This can be done by using the same paradigm with more complex stimuli. This complexity can be increased by the inclusion of consonant clusters, the inclusion of diphthongs and as a result an increase in word length.

Finally, it should be mentioned that there was no no-treatment control group. Although not needed to answer the research questions we had, such a group would provide more information on the general effects of repeated word reading interventions.

### Implications and conclusion

The theoretical implication of this study is that type of feedback offered during repeated word reading does not lead to differential results. Moreover, both good and poor reading children increase their accuracy and speed while repeatedly reading words. Only for pseudowords transfer effects were obtained; hence, it seems that repeated reading of words and pseudowords is especially helpful in training pure decoding skills, i.e., reading via the indirect route. Even though the beneficial effect of phonics feedback was limited, we would suggest to include corrective phonics feedback (so only when an error is made) for poor readers, either provided by a computer or a tutor. Future research could indicate whether feedback only on incorrect items is indeed more effective, since it is likely to be less disturbing for fluency building. The improvement over time through repeated word reading was larger for poor readers than for good readers. In this study, we found that ten repetitions are sufficient to maintain reading speed and accuracy over time, which was measured with a retention task. To help beginning readers with improving their decoding skills, repeated reading of words can thus be helpful.

In sum, we conclude that repeated reading of single words and pseudowords is not affected by the type of feedback that is offered. Apart from that, we can conclude that repeated reading of words is, in general, equally effective for good and poor reading children, since similar retention and transfer effects were found in both groups. Even though direct effects of speed were found for both groups of readers, the poor readers increased the most. Also, the transfer effects were stronger for pseudowords than for words. Based on these findings, it can tentatively be assumed that repeated reading of words mainly strengthens early decoding skills, or letter-by-letter decoding.

## Appendix

**Table 7:** Items used in the experiment. List A was used for the transfer task and List B for the training and retention tasks

List A		List B	
Words	Pseudowords	Words	Pseudowords
gat	gam	gum	gak
gil	gan	kar	kal
kam	kag	kat	ket
kan	kep	kin	kis
kip	kol	kop	kup
kus	kos	lap	lan
lam	lar	lat	lep
lek	lit	les	lom
lip	lut	lus	lop
mep	mas	man	mar
mes	mel	map	mek
mop	mip	mat	mil
mos	mit	mol	mon
mus	muk	mug	mut
pan	pat	nek	nap
pet	pom	pak	pag
pit	pos	pen	pam
put	puk	pil	pes
rat	ret	pot	pok
rol	rip	rem	rop
rug	rup	rok	rut
sap	sak	sop	sat
sok	san	tas	tan
tak	tep	tik	tis
tor	tos	top	tok

(The same materials were used by Van Gorp et al., 2014)
